# Is recreational use of sildenafil a new trend?

**DOI:** 10.1016/j.amsu.2022.104659

**Published:** 2022-09-16

**Authors:** Muhammad Hashir Nazir, Muhammad Ahmad, Saleha Azeem

**Affiliations:** King Edward Medical University, 54000, Lahore, Pakistan

**Keywords:** cGMP, Cyclic Guanosine-monophosphate, ED, Erectile dysfunction, PAH, Pulmonary Arterial Hypertension, HIV/STD, Human Immunodeficiency Virus/Sexually Transmitted Disease

Dear Editor,

Sildenafil is a selective phosphodiesterase type 5 inhibitor which increases cGMP levels by preventing its degradation, thus causing vasodilation and lowering both systemic and pulmonary arterial pressures. This increases blood flow in the penis, which helps achieve and maintain a state of erection. In 1998, Pfizer's developed Viagra® (Sildenafil citrate) was approved by Food and Drug Authority (FDA) as the first oral drug for erectile dysfunction (ED) [1]. Erectile dysfunction is a condition in which a man cannot attain or sustain a penile erection. Before this miraculous drug came into the market, the treatment of ED was limited to surgeries, intraurethral suppositories and injectable therapies [2]. Since the discovery of the use of sildenafil in ED, its popularity and use has soared.

But even after 24 years since its approval by FDA, the question remains unanswered: Is the use of sildenafil worth the risks and addiction associated with it? Multiple studies have shown the increasing recreational use of sildenafil, especially in younger men who preferably use this pharmacotherapy for pleasing their partners rather than treating ED [3,4]. Besides its use in sustaining an erection, it is also an FDA-approved drug for treating pulmonary arterial hypertension (PAH) because of its vasodilation effect. Studies have shown a reduction in the mortality rate in patients of PAH with the use of sildenafil. In the unavailability (resource-limited) of inhaled nitric oxide, the use of sildenafil has also shown improvement in oxygenation in neonates [5]. Though it was approved by FDA as standard therapy for PAH in 2013, it couldn't establish itself as a non-controversial drug. Some studies still advise limiting the use of sildenafil in treating PAH because of its ineffectiveness or adverse effects [6].

Its use today is also quite different from what it was originally meant to employ. The stigma associated with erectile dysfunction is a major reason for its misuse. The easy availability of this inexpensive drug has also contributed to the increasing demand [3]. The use of sildenafil along with other designer drugs e.g. mephedrone, ecstasy, cannabis, cocaine and ketamine in clubs has increased over the past few years [7]. This recreational use of sildenafil primarily by young men is to increase penile rigidity, the satisfaction of partner, and coital frequency. The stress-induced erectile dysfunction and lack of communication among partners also lead to misuse of sildenafil. Sildenafil use is reported to be more common among men who have sex with men (MSM) and also among heterosexuals engaging in insertive anal sex [8].

According to the study conducted by Atsbeha et al., in a sample size of 65 men, recreational use of sildenafil was found to be 66.2% while medicinal use stood at 33.8% [3]. This is represented in Fig. 1.

**Fig. 1:**
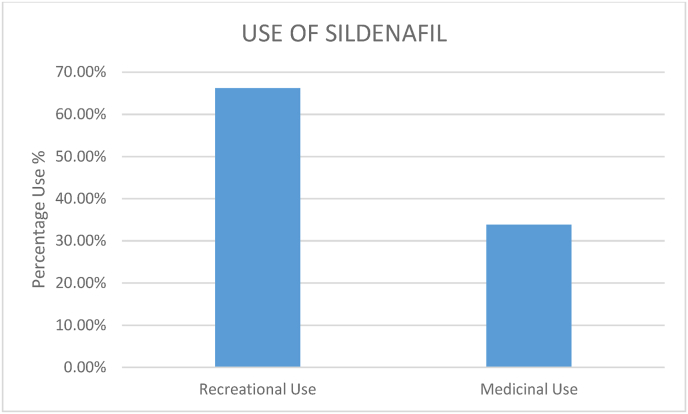
Use of Sildenafil as found in the study by Atsbeha et al., 2021 [3].

This unregulated use of sildenafil comes with its side effects like headache, palpitation and flushing. Sildenafil has a minor inhibitory action on enzyme phosphodiesterase type 6, which is present in rod and cone photoreceptor cells of the retina. This results in changes in the perception of light and colour vision [9]. Potential risks also include a prolonged penile erection lasting more than 4 h, aka priapism, and penile fractures which are medical emergencies. Untreated or delayed-treated priapism can also lead to ischemia [10]. Its misuse and complicated association with rising HIV/STD cases is the cause of its notoriety.

The rising misuse cannot be addressed by removing it entirely from the market. We recommend governments impose regulations on sildenafil use, besides using social mobilizers to raise awareness of its due medicinal use. Making it a prescription drug can play a role in this regard. By banning the illegal sale of Viagra® online through numerous websites, organizing psychotherapy sessions and establishing a standard discipline for sildenafil recommendation, health ministries can partake in preventing its recreational use. Its recreational use can be curbed by educating people about the associated potential risks. We can also opt for talking more openly about ED which can help overcome the stigma associated with it. In conclusion, it is possible and advisable to restrict the recreational use of sildenafil, which is on the rise, particularly among young men.

## Ethical approval

This paper did not involve patients, therefore no ethical approval was required.

## Sources of funding

No funding was acquired for this paper.

## Author contribution

Muhammad Hashir Nazir: conception of the study, literature search, data analysis, final approval and agreeing to the accuracy of the work.

Muhammad Ahmad: literature search, data analysis, participated in writing and agreeing to the accuracy of the work.

Saleha Azeem: Reviewing the letter.

## Registration of research studies

1. Name of the registry: N/A
2. Unique Identifying number or registration ID: N/A
3. Hyperlink to your specific registration (must be publicly accessible and will be checked): N/A

## Guarantor

Muhammad Hashir Nazir.

Muhammad Ahmad.

## Consent

This study was not done on patients or volunteers; therefore, no written consent was required.

## Declaration of competing interest

The authors declare that there is no conflict of interest.
